# Distributions of plantar loads are altered when walking in anxiety-inducing virtual settings but not under cognitive demand

**DOI:** 10.1371/journal.pone.0345075

**Published:** 2026-04-20

**Authors:** Kelly Poretti, Nicole E. P. Stark, Francesca E. Wade, Peter C. Fino, Tiphanie E. Raffegeau

**Affiliations:** 1 Department of Kinesiology, Sport and Hospitality Management, College of Education and Human Development, George Mason University, Manassas, Virginia, United States of America; 2 Department of Biomedical Engineering, Virginia Tech, Blacksburg, Virginia, United States of America; 3 School of Exercise and Nutritional Sciences, San Diego State University, San Diego, California, United States of America; 4 Department of Health and Kinesiology, University of Utah, Salt Lake City, Utah, United States of America; Brunel University London, UNITED KINGDOM OF GREAT BRITAIN AND NORTHERN IRELAND

## Abstract

Additional cognitive load and fall-related anxiety alter balance and locomotor control, independent of motor demands. Exposing participants to virtual elevation increases perceived cost of falling, and individuals alter the distribution of medial and lateral plantar loading to maintain balance and avoid a lateral postural threat (in virtual reality). Here, we extend this prior work to examine anterior and posterior loading in response to cognitive demand and virtual elevation to induce anxiety. We predicted that the distribution of plantar loads between the medial and lateral, and anterior and posterior compartments of in-shoe load sensors would indicate within-step changes to locomotor balance control across walking conditions. Healthy participants (*N* = 16) were pseudorandomized into single- or dual-task trial blocks first. While wearing a head-mounted virtual-reality display and in-shoe load sensors, participants walked at self-selected pace overground on a 40 cm wide by 5.2 m long walkway at virtual high and low elevation during single and dual-tasks. For dual-tasking, participants walked and talked extemporaneously about a randomly selected topic. All participants wore their own footwear. The distribution of medial and lateral and anterior and posterior loads was computed across normalized stance. Statistical parametric mapping determined significant differences in plantar load distribution by walking condition. Fall-related anxiety led to a more medial load distribution during 11% to 75% of stance and more posterior distribution during heel strike from 0% to 4% of stance. There were no load distribution changes due to the extemporaneous speech dual-task. Walking at high virtual elevation, not during a dual-task, led to a medial shift in plantar load during stance, and more posterior loading during initial contact. Plantar load distributions were primarily adjusted within-steps to avoid lateral postural threats when step width was constrained by a narrow path.

## Introduction

Maintaining independence with aging necessitates the performance of multiple tasks simultaneously, such as talking on the phone while walking on a busy sidewalk. Such multitasking requires concurrent motor and cognitive task performance, yet cognitive-motor resources are limited, which leads to performance costs [1], especially in impaired populations [2]. Walking under cognitive load can also change walking function in healthy adults, who usually slow down and take shorter, wider steps during dual-task conditions. [3,4]. Dual-task effect on gait are magnified in older adults [5–7] and people with cognitive-motor function impairments [8–10]. Dual-task interference is particularly evident in the least stable phases of gait, such as single support [11] or weight shift [12], suggesting that attentional demands compromise balance control during locomotion. As compared to the effects of attentional demands, the influence of fall-related anxiety on walking balance remains poorly characterized [13].

When individuals are exposed to postural threat, through real or simulated elevation manipulations, they display reduced sway variability, increased sway frequency (a stiffening response) [14,15], and shifts away from the direction of a perceived threat [14,15]. While these responses play a protective role in quiet standing, they can become maladaptive in dynamic task control, such as effective perturbation recovery or navigating uneven terrain. During walking under virtual elevation, participants reduced walking speed and step length [16–18], altering efficient gait to avoid perceived postural threat [19]. Increased physical task demand, such as fast walking, leads to shorter and more frequent steps [17], a strategy thought to minimize fall-risk [20]. However, the mechanisms underlying threat-induced balance adaptations remain unclear. As locomotor balance reflects both sensorimotor regulation [20,21] and cognitive appraisal of risk [20], examining walking under dual-task and postural threat may clarify how these processes jointly shape balance control.

The center of pressure (COP) provides a key window into how the neuromuscular system regulates locomotor balance. In-shoe load sensors measure plantar loading patterns that align with COP trajectories measured from gold-standard force platforms [22]. During walking, the center of mass (COM) travels medially across the stance foot and shifts toward the future position of the swing limb [23]. To maintain stability, the COP is adjusted to ensure the COM remains within the base of support. Between steps, foot placement adjustments shift the COP, reflected by altered step length and width. Foot placement alterations are clearly demonstrated in both dual-task studies [10,11,24] and in anxiety-inducing settings [13,16,25,26]. Within steps, balance can be maintained by ankle or hip torques that modulate COP beneath the stance foot [27]. While these within step strategies have been investigated under perturbations [28], their modulation under dual-task demands and postural threat remains largely unexplored.

Preliminary work has suggested that anxiety-inducing conditions shift plantar loads medially around mid-stance, consistent with a stiffening strategy while cognitive demands showed no effects [29]. However, prior findings were limited by a small sample size (*N* = 8) and did not include temporal measures such as double support time. The present study builds on this work by systemically examining locomotor balance control under two distinct challenges (1) cognitive load (dual-task), and (2) postural threat. To model everyday dual-task walking, we required participants to speak extemporaneously while walking – a continuous, socially meaningful, and cognitively demanding task that has been shown to slow gait and shorten steps in young adults [26,30]. Difficulty walking and talking is also linked to older adult fall-risk [31] and the extemporaneous speech dual-task reduced propulsive force and COP anterior-posterior velocity in older adults [32]. To impose fall-related anxiety without introducing actual risk, we used virtual reality (VR) to simulate an ~ 15 m elevation above ground, an approach known to elevate state anxiety during standing [14], walking [13,26], and turning [20,33] in young and older adults.

Based on prior findings of postural threat [14,20] and pilot work on plantar load distribution [29], we predicted that dual-task demands and fall-related anxiety, alone and in combination, would alter within-step balance regulation. Specifically, we expected plantar loads to reflect a more medial COP trajectory and constrained anterior and lateral excursions, consistent with a stiffening response that reduces lateral COM motion [20]. To contextualize these findings, we also assessed spatiotemporal gait outcomes (speed, step length, step width, and double support; Appendix A).

## Materials and methods

### Participants

We included participants free of any injury or disorder that may impact their walking or comfort using VR. Participants were required to be between the ages of 18 and 35 and have hearing and vision corrected to normal. Participants were excluded if they reported neurological, orthopedic, or cardiovascular conditions, or excessive motion sickness or vertigo that could interfere with their use of VR. Recruitment occurred between November 3^rd^, 2023, and November 8^th^, 2024, with course credit provided as incentive. All participants gave informed written consent; procedures were approved by the University’s Institutional Review Board (approval #1979005), in accordance with the Declaration of Helsinki.

### Procedures

Participants wore load-sensing insoles (Loadsol Pro, novel electronics inc., Pittsburgh, PA) inside their own shoes. These wireless sensors sampled vertical loads from the medial and lateral forefoot, as well as from the rearfoot, at 100 Hz. Rear-forefoot segmentation occurred at 40% of the foot length from the heel. For the virtual reality exposure, participants wore a head-mounted display (HTC Vive 2.0, Bellevue, WA), which presented the virtual environment and ambient sounds (i.e., birds chirping, running water) to enhance immersion.

**Fig 1:**
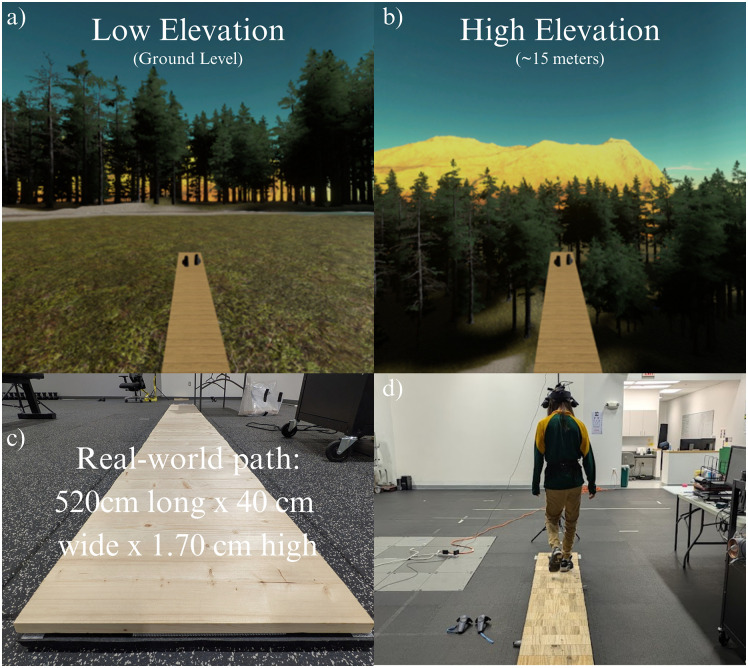
a-d. Virtual and real-world walking environments. (a) Ground-level (virtual low) height, (b) Elevated (virtual high) condition, at 15 m above ground level. (c) Real walkway to which VR dimensions were matched (d) Participant walked while wearing a commercially available wireless head-mounted VR system.

Walking trials consisted of two single (ST) conditions (Fig 1a-b): 1) ground level (virtual low) and 2) 15m above ground (virtual high). The VR walkway measured 0.4 x 5.2 m and matched a real, wooden plank path (Fig 1c-d), as in prior work [26]. Each condition lasted one minute at self-selected speed. To complete a loop, participants performed 180° turns (in either direction), though those steps were excluded from analysis.

Compared with the previous pilot work [29], the VR environment was simplified: asymmetric features such as waterfall and flowing stream, and the dusk-like lighting used previously were removed, leaving a uniformly forested setting. While this reduced environmental realism, it minimized optic flow disturbance and environmental distraction.

For dual-task blocks, the walking set-up was identical, but participants also performed an extemporaneous speech task. At the start of a dual-task block, participants completed this speech trial while seated, as a single task. They selected six topics from a list of 26 (e.g., describe your first job, pets, sports played, travel experiences, a room in your home) and spoke continuously for one minute per topic. Participants were reminded that the content of their speech was irrelevant; the goal was sustained speech.

Participants completed one, one-minute trial in each condition (low elevation ST, high elevation ST, low elevation DT, high elevation DT) for a total of four trials. Task order (ST or DT) was randomized, but to equate fall-related anxiety levels across conditions [34], all participants performed the virtual low condition before the virtual high. After each condition, participants rated cognitive and somatic anxiety and task confidence using the Mental Readiness Form-3 (MRF) [35]. Participants reported mental effort dedicated to the task using the Rating Scale of Mental Effort (RSME) [36].

### Plantar load analysis

**Fig 2:**
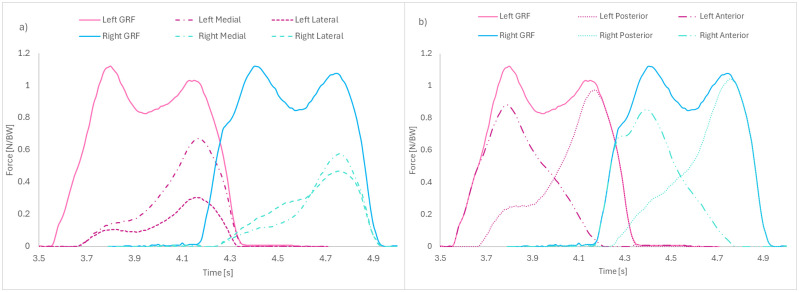
a-b. Plantar Load Distributions in the Medial and Lateral (a) and Anterior and Posterior (b) Regions. Loadsol sensors measured the vertical loads in three separate compartments (medial, lateral, and heel) in the left and right feet. **(a)** Medial loads are represented by the dashed and dotted line, lateral loads are represented by the dashed line, and total loads representative of net Ground Reaction Forces (GRF) are a summation of the medial, lateral, and heel loads and are represented by the solid lines. **(b)** Anterior loads are a summation of the medial and lateral loads and are represented by a dotted line, posterior loads are represented by the dashed and double dotted line, and total loads are represented by solid lines.


Loadsol data provided medial, lateral, and heel vertical force signals (Fig 2), and were extracted for step analysis using a publicly available visualization interface [37] that was modified for the Loadsol three-compartment signal in MATLAB (v2022b). Loads were normalized to body weight and time normalized across 0–100% stance. Analysis focused on the first 19 steady-state steps, ensuring the largest equal step count across participants. Relative medial and lateral plantar load distributions were examined by calculating the difference between the lateral and medial loads, where:

$$ML\;Plantar\;Load\;Distribution=\;\frac{\left({Load(N)}_{lateral}-{Load(N)}_{medial}\right)}{BW(N)}$$

*Variables load(N) and bodyweight (BW) are expressed in Newtons.

Relative anterior and posterior plantar load distributions were examined by calculating the difference between the rear load and the combined load of the medial and lateral plantar loads, where:


$$AP\;Plantar\;Load\;Distribution=\;\frac{\left(Load(N{)}_{heel}-[Load{\left(N\right)}_{medial}+Load{\left(N\right)}_{lateral}\right)}{BW(N)}$$

*Variables load(N) and bodyweight (BW) are expressed in Newtons.

A larger plantar load distribution represents a posterior or lateral COP shift while a smaller value reflects an anterior or medial COP position on the stance foot [22]. These indices quantify within-step balance adjustments, whereas between step control is captured by spatiotemporal outcomes.

### Spatiotemporal gait measures

Using 3D positional data from ankle trackers (HTC Vive, v2) and the headset, we calculated step length, step width, gait speed, and double support time (%), using a custom MATLAB code, which are reported in Supplementary Data Tables. We identified gait events from changes in the vector-quantified headset-to-ankle distance (toe-off: minimum; initial contact: maximum). Steps during gait initiation and termination were excluded, following prior recommendations [38], while turning steps were eliminated using the visual interface mentioned previously [37]. We calculated step length as the anterior-posterior distance between contralateral heel strikes, averaged across limbs, while step width represented the mediolateral distance between contralateral heel strikes, averaged across limbs [23]. Gait speed per stride was found as the anterior-posterior distance between ipsilateral, subsequent heel strikes divided by the amount of time between these heel strikes [23]. Gait cycle time was the time between ipsilateral heel strikes [23]. Finally, we calculated double support per gait cycle, measured as the sum of time from heel strike to contralateral toe-off bilaterally, normalized to cycle duration [23].

### Statistical analysis

Differences in plantar load distribution across stance were tested using one-dimensional Statistical Parametric Mapping (SPM), which determines the regions of difference using suprathreshold clusters [39]. Two separate two-way (factorial) repeated measures SPM analyses examined effects of virtual height (2 levels: Low (reference), High) and task (ST(reference), DT) on 1) mediolateral and 2) anterior posterior load distributions (α = 0.05). Linear mixed effects regression (LMER) models compared MRF and RSME scores, as well as spatiotemporal gait parameters (see Appendix A), across the same factors. The significance threshold for all analyses was set at α  =  0.05 and performed in R studio (Version 2023.09.0).

*A priori* power analysis was conducted with pilot data [29], following documentation provided by the power1d package in Python that was developed to calculate power for one-dimensional continual data as used in this study [39]. Pilot study data [29] achieved a power of  .890 for an *N* of 13, and  .920 for an *N* of 14. Based on these estimates, we recruited 16 participants, which provided adequate power for this analysis.

## Results and discussion

Participant demographics (*N* = 16, 12 women) are reported in Table 1. Spatiotemporal gait outcomes are reported in Appendix A (Supplementary Data Table 1). Loadsol data are available in supporting documents.

**Table 1:** Participant Characteristics (N = 16).

	Mean	*SD*	Range	
			Lower limit	Upper limit
Age (y)	23.1	3.3	18.7	31.9
Height (cm)	165.0	8.9	150.0	182.6
Mass (kg)	73.0	10.8	55.1	92.9
Leg length (cm)	88.9	5.4	82.1	101.9
Gender (women/men)	12/4	--	--	--

Note: SD = standard deviation; y = year.

### Self-report outcomes

Our results revealed main effects of Height for cognitive anxiety (*p* < .001), somatic anxiety (*p* < .001), confidence (*p* < .001), and mental effort (*p* < .001) (Table 2). Participants reported greater levels of worry, tension, and mental effort, along with lower levels of confidence in their ability to perform the task when walking at high VR heights compared to low VR heights. Our results revealed an interaction effect (*p* = .026) for mental effort, where participants expressed significantly greater levels of mental effort at high VR heights compared to low VR heights during ST (*p* < .001) and DT conditions (*p* < .001).

**Table 2:** Fixed effects of Height, Task, and Height*Task for self-reported cognitive anxiety, somatic anxiety, confidence, and mental effort.

Fixed Effects	Low ST Mean(SD)	High ST Mean(SD)	Low DT Mean(SD)	High DT Mean(SD)	*β*	*df*	*F*	*p*
**Cognitive Anxiety**	3.06(1.9)	7.19(2.4)	2.44(1.4)	5.40(2.6)				
Intercept					3.1	60	24.27	**<.001**
Height					4.1	60	29.67	**<.001**
Task					−0.6	60	2.24	.311
Height*Task					−0.9	60	11.61	.149
**Somatic Anxiety**	3.56(1.8)	6.94(2.3)	3.06(1.8)	5.53(2.9)				
Intercept					3.6	60	30.56	**<.001**
Height					3.4	60	26.79	**<.001**
Task					−0.5	60	1.18	.448
Height*Task					−0.8	60	13.66	.091
**Confidence**	2.75(1.7)	5.69(2.5)	2.44(1.5)	4.47(2.3)				
Intercept					2.8	60	21.42	**<.001**
Height					2.9	60	22.03	**<.001**
Task					−0.3	60	0.70	.529
Height*Task					−0.7	60	7.95	.158
**Mental Effort**	28.13(22.6)	68.75(26.8)	32.50(12.4)	59.33(24.0)				
Intercept					28.1	60	19.59	**<.001**
Height					40.8	60	39.38	**<.001**
Task					4.38	60	1.34	.465
Height*Task					−13.0	60	23.46	**.026**

### Plantar load distributions

**Fig 3:**
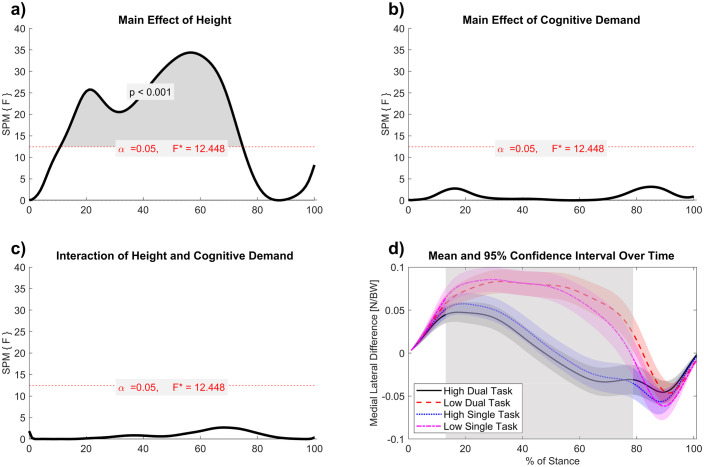
a-d. Medial–lateral plantar load distribution mean curves with 95% confidence intervals, with results of the SPM two-way repeated-measures ANOVA. Significant cluster revealed for main effect of height (Low (reference), High) (a). No significant clusters were detected for the main effect of cognitive demand (Single task (reference), Dual Task) (b) and the interaction of VR height and cognitive demand (c). Difference values indicate difference between medial and lateral normalized vertical loads (Newtons divided by Body Weight) (d). A larger value represents a more lateral COP shift, a smaller value a more medial COP shift. The grey shaded region indicates the significant cluster for the main effect of VR height, where in high VR height increased medial load bias between 11% and 75% of stance (*p* < .001).

SPM two-way repeated measures ANOVA for the medial and lateral plantar load distribution revealed a region of difference for the main effect of height from 11% to 75% of stance (*F* = 12.448, *p* < .001) (Fig 3). The medial and lateral plantar load distribution was decreased at high VR height compared to low VR height, indicating a more medial position of the COP (Fig 3). Descriptively, the medial and lateral plantar load distribution at high heights was a smaller range (−0.05 to 0.05) than at low heights (−0.05 to 0.1). There were no regions of significant difference in medial and lateral plantar load distribution for the main effect of cognitive demand, nor the interaction between cognitive demand and height.

**Fig 4:**
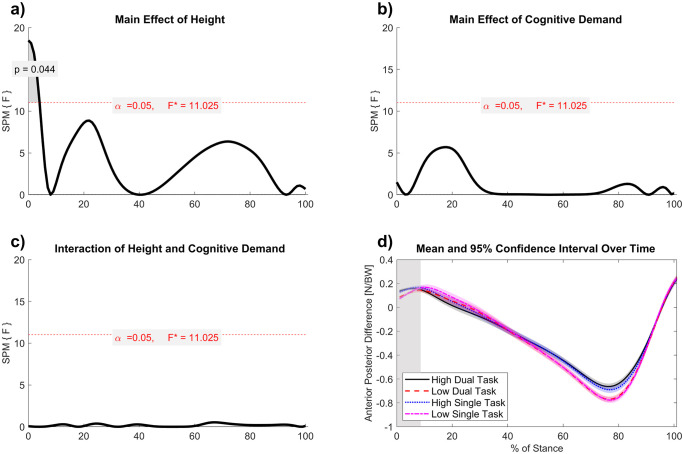
a-d. Anterior and posterior plantar load distribution mean curves with 95% confidence intervals, with results of the SPM two-way repeated-measures ANOVA. Significant cluster revealed for main effect of height (Low (reference), High) **(a)**. No significant clusters were detected for the main effect of cognitive demand (Single task (reference), Dual Task) (b) and the interaction of VR height and cognitive demand **(c)**. Difference values indicate difference between anterior (medial + lateral) and rear normalized vertical loads (Newtons divided by Body Weight) **(d)**. A larger value represents a more posterior COP shift, a smaller value a more anterior COP shift. The grey shaded region indicates the significant cluster for the main effect of VR height, where in high VR height increased medial load bias during 0% to 4% of stance (*p* = .044).

The SPM two-way repeated measures ANOVA for the anterior and posterior plantar load distribution revealed a minor region of difference for the main effect of height from 0% to 4% of stance (*F* = 11.025, *p* = .044) (Fig 4). The anterior and posterior plantar load distribution was briefly increased at high VR height compared to low VR height, representing a more posterior position of the COP at approximately 0–4% of the stance phase (Fig 4). There were no regions of significant difference for the main effect of cognitive demand, nor the interaction of cognitive demand and height. Descriptively, the distribution of anterior and posterior plantar loads followed a similar pattern and range across all tasks.

## Discussion

We investigated how balance control strategies during walking are influenced when individuals face both cognitive demands and heightened postural threat. Using immersive VR, we successfully elicited fall-related anxiety, as evidenced by increasing self-report anxiety at elevation compared to ground level. While performing continuous speech at both heights, we measured within-step plantar load distributions to capture changes in the control of mediolateral and anteroposterior balance. Consistent with earlier findings on gait analysis under postural threat [29], participants shifted plantar loads medially, with reduced variability across stance, and showed a more posterior loading pattern at heel strike in high-anxiety conditions. However, we observed these medial shifts across a broader portion of stance than previously revealed, extending well beyond the isolated 34% phase previously reported. Contrary to expectations, but in alignment with previous work [26,29] concurrent cognitive demand did not elicit detectable changes in plantar loading, suggesting that anxiety-driven adaptations are prioritized over dual-task adjustments in steady-state walking. Our results highlight that destabilizing influences are not uniformly expressed across the gait cycle, but instead emerge during critical transitional phases, i.e., during single support and at heel strike, where control of the COM relative to the COP is most challenging.

The medial redistribution of plantar loads under fall-related anxiety likely reflects a conservative strategy to constrain COM excursions. Consistent with the observed shifts in plantar loading, participants also exhibited slower gait speeds, shorter step lengths, and longer double support times at elevated virtual heights and during dual-task conditions. Such spatiotemporal adjustments are characteristic of a cautious gait strategy, reflecting an effort to enhance postural stability by increasing the proportion of the gait cycle spent in double support and reducing dynamic COM excursions. While traditional cautious gait frameworks predict lateral COP shifts to increase margins of stability, our results suggest that participants instead constrained frontal plane accelerations through a medial COP shift. Descriptive trends toward narrower step widths reinforce this interpretation of a stiffening control strategy, where minimizing COM velocity may be more important than maximizing step-to-step margins. Comparable adaptations have been observed when walking at high virtual elevations, where step narrowing reduces lateral velocity and perceived fall-risks [20]. Future work should quantify the COM-COP relationships more directly, and incorporate mechanical stability metrics, such as margin of stability [40,41].

Within step adjustments that shift the COP are often complemented by changes in foot placement strategy to maintain mechanical stability [42]. In addition to mediolateral adjustments, posterior shifts in plantar loading at heel strike were observed under height-induced anxiety. Anterior-posterior adaptations may be related to slower speeds, or reflect a coupling between slower gait speeds, shorter step lengths, and anticipatory control. The posterior redistribution of plantar loads at heel strike and prolonged double support intervals may act in concert to reduce forward momentum and facilitate anterior-posterior corrective control when balance demands are elevated. Since effective forward progression requires the COP to be positioned posterior to the COM and extrapolated COM [40,41], a backward shift at heel strike may enable individuals to better regulate deceleration, potentially supporting rapid stopping or corrective maneuvers. While a direct comparison of COM-COP mechanics is outside the scope of this paper, overall, testing this possibility with gait termination paradigms could clarify whether such posterior shifts serve as protective mechanisms under threat.

In late stance (75–100%), plantar load distribution resembled that at low heights, suggesting that when both feet contribute to stability (as in double support), the need for COP adjustments diminishes (Fig 3d). Typically, COP progression transitions from the heel to the lateral forefoot (1^st^ metatarsal) before shifting medially to initiate swing [23]. Anticipatory lateral torques at push-off often fluctuate (“over-” and “under-shooting” the COM) to maintain stability, interdependently adjusting stability with foot placement [27]. In fall-anxiety inducing contexts, adopting lateral loading during push-off may compensate for earlier medial shifts, enabling smoother transfer between limbs [44]. Our findings reinforce that foot placement remains a dominant stability mechanism [27,28,43], but that plantar pressure distributions demonstrate an additional, flexible means of modulating stability when facing environmental threat.

Dual-task conditions did not meaningfully alter plantar load distribution, agreeing with results from an initial pilot study [29]. Participants altered spatiotemporal parameters during an extemporaneous speech dual-task as predicted and in alignment with previous studies where healthy participants prioritized extemporaneous speech and allowed gait to change [3,26]. Despite the presence of threat, healthy participants still prioritize speaking, perhaps due to social consequences of failure, supported by similar levels of silent speech pauses (>150ms) from seated speech to walking at low and high elevation [26]. In this study and in and initial pilot analysis [29], the lack of significant change in the plantar load distribution during the dual-task suggests a balance-focused stepping strategy that is predominant only under fall-related anxiety, perhaps due to the spatial constraints of avoiding a fall at high elevation. Possibly, the verbalization aspect of the cognitive task may have acted as a distraction from height-related anxiety, as previously interpreted experimentally [26,44] and in alignment with theories of attentional control under anxiety [45,46]. Additionally, available speech topics are designed so that individuals can speak extemporaneously and continuously for the entirety of the walking trial [47], but one might argue some of the topics participants selected may have led to a positive affect (such as describing pets), further distracting from anxiety. Although neutral topics such as ‘describing a room in your home’ are available topics to select, those topics are rarely- if ever- selected by participants as easily spoken about for a minute. Refined analysis of the valence of the speech topic is outside of the scope of the current study, but future studies should more closely examine topic-specific dual-task costs to extemporaneous speech pauses and syllabic complexity. Further, our choice of dual-task is closer to real-word dual-tasking and imposes a continuous demand compared with other cognitive dual-tasks, such as verbal fluency tests, and does not cause any sensory interference, such as visual Stroop tasks, which may limit generalizability of our results to other types of dual-task.

### Limitations and future directions

Although this study provides intriguing insights into step-to-step regulation of balance during anxiety-inducing gait, several limitations warrant caution. First, we included a homogenous sample of young, healthy individuals (*N* = 16, Table 1), limiting the generalizability of our results to older or mobility-impaired populations in whom anxiety effects may be more pronounced. Although the in-shoe load sensors offer portability and ecological validity [33], fitting across diverse foot sizes with only three pairs of insole may have reduced sensitivity to subtle individual differences. Because the in-shoe load sensors record vertical loads in three discrete regions (medial and lateral forefoot, and rearfoot) rather than providing a continuous plantar pressure map, we were unable to capture localized peak pressure distributions across the full foot surface. We also chose to allow participants to wear their own comfortable walking shoes rather than standardized footwear; while this may increase variability and reduce sensitivity, it increases the generalizability of our findings to real-world footwear conditions [37]. Additionally, only one dual-task condition involving extemporaneous speech was evaluated in this study. The speech task was selected to reflect a continuous naturalistic verbal–motor challenge commonly encountered during real-world walking. However, this choice limits generalization to other forms of cognitive load that future work can explore.

Our long-term goal is to integrate plantar load monitoring into a commercial-VR paradigm for use in the community, where portable tools could aid in identifying fall risk and guiding rehabilitation for individuals at risk of falling without access to conventional rehabilitation. Finally, advanced analytic methods, including SPM, should be applied in future work to capture nuanced temporal shifts in load distributions across populations with greater fall risk.

## Conclusion

Walking in fall anxiety-inducing VR settings resulted in distinct within-step adaptations in plantar loading, with medial shifts during single support and posterior shifts at heel strike. Altering loading patterns suggests that under postural threat, individuals adopt conservative strategies to constrain lateral COM motion, relying on COP adjustments to complement foot placement. In healthy adults, such responses appear adaptive, but older adults may need to rely on different neuromuscular control strategies as a result of altered lower limb torque coordination [48]. The extent to which older adults or mobility-impaired individuals employ similar mechanisms remains unknown and warrants future study. Clarifying the interplay between the COP-COM control in vulnerable populations will be essential for understanding fall-related anxiety and informing targeted interventions.

## Supporting information

S1 File Dataset. Study data is available here.
(ZIP)

S2 File Supplementary Data. Supplementary data analysis of electrodermal activity.
(DOCX)
