# Supplementary material for: Distributions of plantar loads are altered when walking in anxiety-inducing virtual settings but not under cognitive demand
Source: PLoS One. 2026 Apr 20;21(4):e0345075. doi: 10.1371/journal.pone.0345075 (PMC13094994; doi:10.1371/journal.pone.0345075)
Supplement: S2 File — Supplementary data analysis of electrodermal activity. (DOCX) [file pone.0345075.s002.docx]

S2 File. **Supplementary Data.** Supplementary data analysis of spatiotemporal gait outcomes.

S2. **Supplementary Data.** Supplementary data analysis of spatiotemporal gait outcomes.

**Supplementary Data Table 1: Fixed effects of Height, Task, and Height*Task for spatiotemporal gait parameters.**

| **Fixed Effects** | **Low ST Mean(SD)** | **High ST Mean(SD)** | | **Low DT Mean(SD)** | **High DT Mean(SD)** | ***β*** | ***df*** | ***F*** | ***p*** |
| --- | --- | --- | --- | --- | --- | --- | --- | --- | --- |
| **Step Length** | .53(.1) | .46(.1) | | .49(.1) | .45(.1) |  |  |  |  |
| Height |  |  | |  |  | -.057 | 16.6 | 8.920 | **.008** |
| Task |  |  | |  |  | -.033 | 9.4 | 9.940 | **.011** |
| Height*Task |  |  | |  |  | .017 | 2682.2 | 7.353 | **.007** |
| **Step Width** | 0.23(.04) | .23(.03) | | .23(.04) | .22(.03) |  |  |  |  |
| Height |  |  | |  |  | -.003 | 15.4 | 3.964 | .065 |
| Task |  |  | |  |  | .003 | 30.8 | .169 | .684 |
| Height*Task |  |  | |  |  | -.002 | 2261.0 | 1.238 | .266 |
| **Gait Speed** | 0.86(.2) | | 0.73(.2) | 0.75(.2) | 0.69(.2) |  |  |  |  |
| Height |  |  | |  |  | -.082 | 9.0 | 12.642 | **.006** |
| Task |  |  | |  |  | -.095 | 16.6 | 4.389 | .052 |
| Height*Task |  |  | |  |  | .052 | 2683.8 | 19.823 | **<.001** |
| **Double Support Time** | .25(.04) | .28(.06) | | .27(.04) | .28(.05) |  |  |  |  |
| Height |  |  | |  |  | .017 | 26.6 | 2.364 | .136 |
| Task |  |  | |  |  | .013 | 13.3 | 13.566 | **.003** |
| Height*Task |  |  | |  |  | .007 | 2382.0 | 4.971 | **.026** |

***Spatial Gait Parameters***

Our results revealed main effects of Height, *F*(1, 16.6) *=* 8.920*, p* = .008, *β* = -.057 m, [95% Confidence Interval (CI) = -.097, -.018], and Task *F*(1, 9.4) *=* 9.940*, p* = .011, *β* = -.033 m, [CI = -.056, -.010], as well as an interaction effect *F*(1, 2682.2) *=* 7.353*, p* = .007, *β* = .017 m, [CI = .005, .029], for step length. Participants took significantly shorter steps than the mean step length during dual-task conditions at both low *F*(1,1320.6) = 81.800, *p* < .001, and high *F*(1,1367.3) = 30.611, *p* < .001, virtual heights compared to single-task conditions. Additionally, participants took significantly longer steps at low virtual heights compared to high virtual heights and during dual-task conditions compared to single-task conditions. No significant main effects of Height (*p* = .065) or Task (*p* = .684), nor interaction effects (*p* = .266) were revealed for step width.

***Temporal Gait Parameters***

Our results revealed an interaction of Height and Task for gait speed, *F*(1, 2683.8) = 19.823, *p* < .001, *β* = .052 m/s, [CI = .029, .074], and double support time, *F*(1, 2382.0) = 4.971, *p* = .026, *β* = .007%, [CI =-.013, -.001]. Our results revealed main effects of Height for gait speed, *F*(1, 9.0) *=* 12.642*, p* = .006, *β* = -.082 m/s, [CI = -.159, -.004], but not for double support time (*p* = .136). Participants took significantly slower steps than the mean gait speed at high settings compared to low settings during single-task conditions, *F*(1, 1313.5) *=* 183.990*, p* < .001, *β* = -.113 m/s, and during dual-task conditions, *F*(1, 1371.6) *=* 55.419*, p* < .001, *β* = -.060 m/s. Main effects of Task were revealed for double support time, *F*(1, 13.3) *=* 13.566*, p* = .003, *β* = .013 m/s, [CI = .006, .021], but not for gait speed (*p* = .052). Participants spent significantly more time in double support during dual-task conditions at low settings *F*(1, 1188.5) *=* 93.103*, p* < .001, *β* = .021%, and at high settings *F*(1, 1239.0) *=* 42.130*, p* < .001, *β* = .014%, compared to single-task conditions.
